# Prevotella denticola Bacteremic Spondylodiscitis and Epidural Abscess Associated With a Periapical Lesion: A Case Report

**DOI:** 10.7759/cureus.112118

**Published:** 2026-07-06

**Authors:** Yuji Noda, Kenji Noda, Shu Takahashi, Takao Sato

**Affiliations:** 1 Emergency Medicine, Asao General Hospital, Kawasaki, JPN; 2 Dentistry, Noda Dental Clinic, Shibuya, JPN; 3 Orthopedics, Asao General Hospital, Kawasaki, JPN

**Keywords:** bacteremia, epidural abscess, odontogenic infection, prevotella denticola, spondylodiscitis, vertebral osteomyelitis

## Abstract

*Prevotella denticola *is an anaerobic gram-negative rod that commonly inhabits the oral cavity and is associated with periodontal and periapical infections. Vertebral osteomyelitis caused by *Prevotella* species is uncommon, and reports involving* P. denticola* are exceedingly rare.

A 65-year-old man presented with progressive low back pain and difficulty walking following multiple falls. Lumbar magnetic resonance imaging demonstrated L2-L3 spondylodiscitis with an epidural abscess. Fluoroscopy-guided disc aspiration and subsequent full-endoscopic spinal surgery with percutaneous endoscopic discectomy and drainage were performed. Both blood cultures and disc aspirate cultures yielded *P. denticola*. Further investigation by dental specialists revealed a periapical lesion and periodontal disease, suggesting an odontogenic source of bacteremia. The patient was successfully treated with surgical source control and prolonged antimicrobial therapy, resulting in resolution of inflammatory markers and clinical improvement.

This case highlights the importance of considering anaerobic odontogenic pathogens as potential causes of hematogenous vertebral infections. Comprehensive source investigation, including dental evaluation, should be considered when anaerobic organisms are isolated from blood or spinal specimens.

## Introduction

Native vertebral osteomyelitis is an uncommon but potentially devastating infection that often results from hematogenous bacterial dissemination [1-3]. *Staphylococcus aureus* remains the most frequently identified pathogen; however, anaerobic organisms account for only a small proportion of reported cases [4,5]. 

*Prevotella* species are anaerobic gram-negative bacilli that form part of the normal oral microbiota and are frequently implicated in periodontal disease, periapical abscesses, and other odontogenic infections [6-8]. Although transient bacteremia associated with oral infections and dental procedures is well recognized, vertebral osteomyelitis caused by *Prevotella intermedia* [9-11], *P. melaninogenica* [12], and *P. oralis* [13], as well as spinal infections secondary to dental procedures or dental abscesses [14,15], have only rarely been reported.

We report a case of bacteremic spondylodiscitis and epidural abscess caused by *P. denticola* in which both blood cultures and spinal cultures were positive, and dental imaging identified a probable odontogenic source. 

## Case presentation

A 65-year-old man presented to our hospital with worsening low back pain and progressive difficulty walking.

His medical history was significant for cerebral infarction, intracerebral hemorrhage, myocardial infarction, hypertension, and dyslipidemia. He had no history of diabetes mellitus, malignancy, chronic immunosuppressive therapy, or other known immunocompromising conditions.

Approximately nine days before admission, he experienced multiple falls, including one fall down approximately five stairs. He initially sought medical attention elsewhere and was managed conservatively. However, persistent low back pain and impaired ambulation prompted referral to our institution.

On admission, his temperature was 36.6°C, blood pressure was 195/66 mmHg, heart rate was 66 beats/minute, and oxygen saturation was 98% on room air. Physical examination revealed marked tenderness over the lumbar spine. No focal neurological deficits, lower extremity weakness, or bowel/bladder dysfunction were present.

Initial laboratory studies demonstrated a white blood cell count of 11,270/μL and a C-reactive protein (CRP) level of 3.50 mg/dL. During hospitalization, CRP increased to a peak value of 7.64 mg/dL.

**Figure 1:**
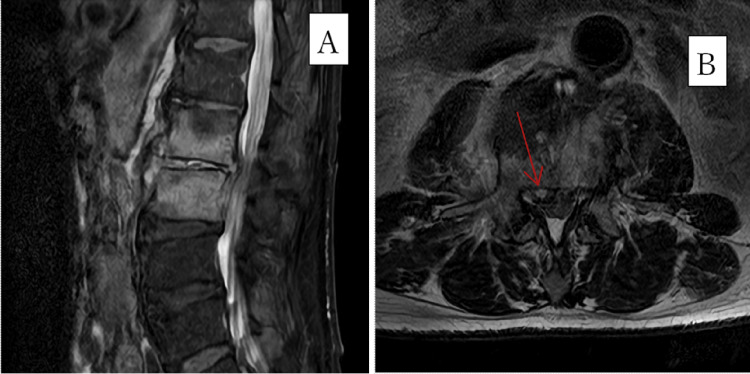
Magnetic resonance imaging findings of lumbar spondylodiscitis and epidural abscess (A) Sagittal magnetic resonance imaging of the lumbar spine demonstrating abnormal signal intensity involving the L2-L3 intervertebral disc and adjacent vertebral endplates, consistent with spondylodiscitis. Endplate destruction and inflammatory changes are evident. (B) Axial contrast-enhanced magnetic resonance imaging demonstrating an epidural fluid collection (arrow) at the L3 level, compatible with an epidural abscess.

Lumbar magnetic resonance imaging demonstrated signal abnormalities involving the L2-L3 intervertebral disc and adjacent vertebral endplates, consistent with spondylodiscitis. An associated epidural abscess was identified at the L3 level (Figures 1A, 1B).

**Figure 2:**
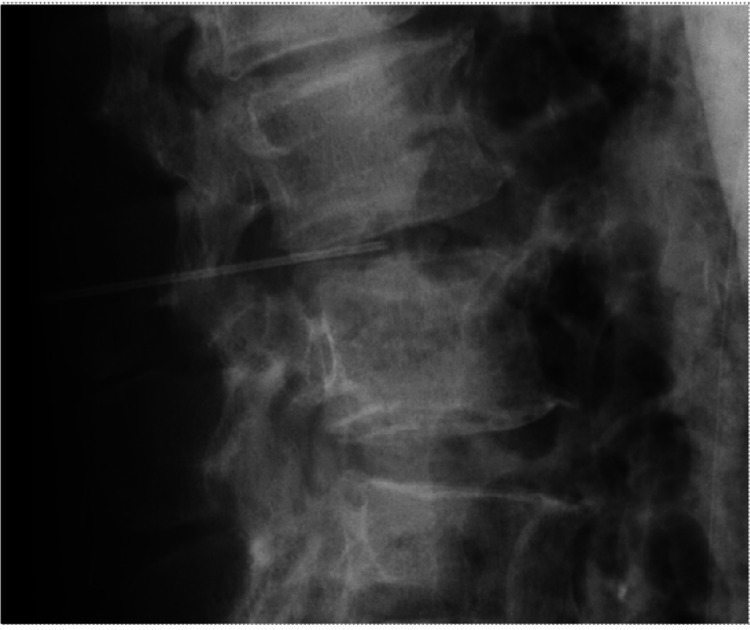
Fluoroscopy-guided disc aspiration for microbiological diagnosis Fluoroscopic image obtained during needle aspiration of the infected L2-L3 intervertebral disc space. The procedure was performed to obtain specimens for microbiological diagnosis of suspected spondylodiscitis. Culture of the aspirated specimen subsequently yielded *Prevotella denticola.*

Fluoroscopy-guided disc aspiration was performed on hospital day 5, and specimens were submitted for microbiological analysis (Figure 2). Empirical cefazolin therapy was initiated following the procedure.

Because of persistent deep spinal infection despite initial treatment, full-endoscopic spinal surgery/percutaneous endoscopic discectomy and drainage (FESS/PEDD) was performed on hospital day 7. Intraoperatively, necrotic and inflammatory tissue was identified within the infected disc space, and extensive irrigation was performed. To alleviate pain associated with vertebral instability, a rigid thoracolumbar brace was applied. The brace was well tolerated and allowed gradual mobilization during hospitalization.

Empirical intravenous cefazolin (1 g every eight hours) was initiated following fluoroscopy-guided disc aspiration. Subsequently, *P. denticola* was identified from both blood cultures and disc aspirate cultures, confirming the diagnosis of bacteremic spondylodiscitis. Antimicrobial therapy was modified to pathogen-directed treatment. Intravenous ceftriaxone (2 g once daily) was administered, followed by intravenous ampicillin/sulbactam (3 g every six hours). After clinical improvement, the patient was transitioned to oral amoxicillin/clavulanate. Intravenous antimicrobial therapy was continued for approximately six weeks, followed by two weeks of oral therapy, for a total treatment duration of eight weeks.

**Figure 3:**
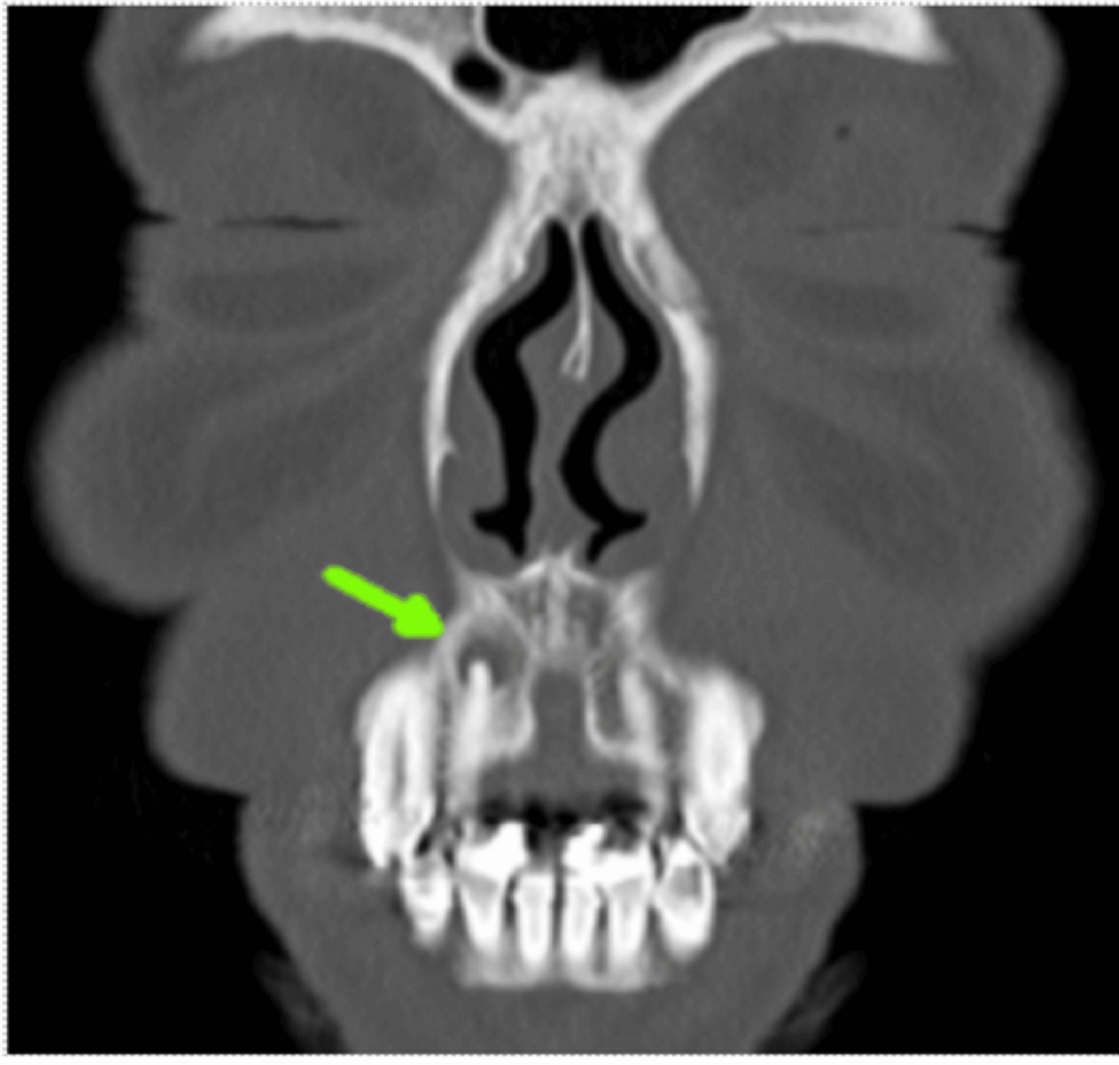
Dental computed tomography demonstrating the presumed odontogenic source of bacteremia Dental computed tomography revealed a periapical cystic lesion associated with the right maxillary lateral incisor (arrow). The crown was fractured, and the tooth was non-vital. In addition, generalized alveolar bone resorption involving both the maxilla and mandible was observed, consistent with chronic periodontitis. These findings were considered the most likely source of *Prevotella denticola *bacteremia.

To identify the source of bacteremia, a comprehensive evaluation was performed. Dental consultation and dental computed tomography revealed a periapical radiolucent lesion involving the right maxillary lateral incisor and evidence of chronic periodontal disease (Figure 3). These findings were considered the most likely source of hematogenous dissemination. 

**Figure 4:**
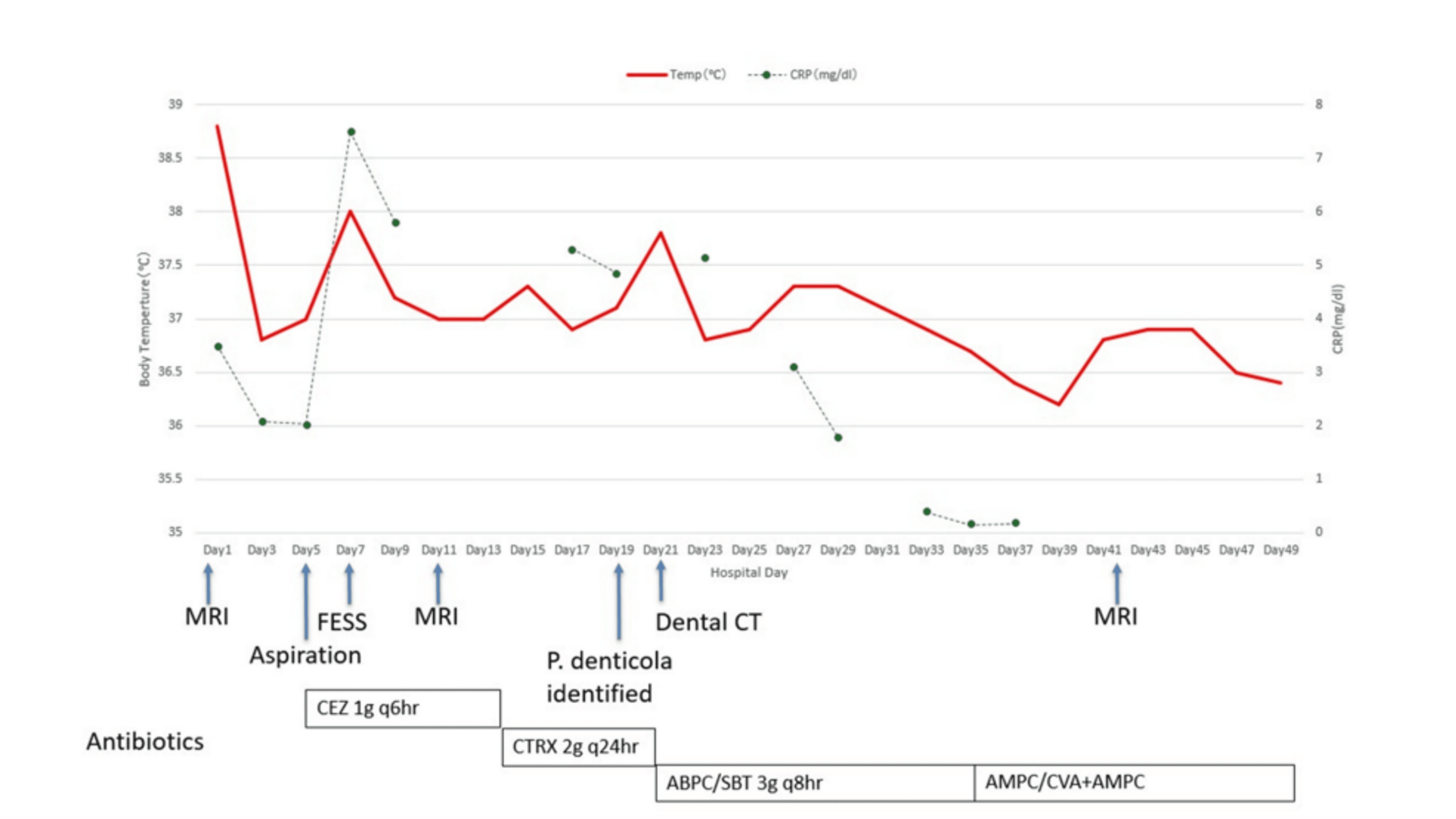
Clinical course, inflammatory markers, microbiological findings, and antimicrobial therapy The patient's clinical course is shown. Lumbar magnetic resonance imaging performed on hospital day 1 demonstrated L2-L3 spondylodiscitis with an epidural abscess. Fluoroscopy-guided disc aspiration was performed on hospital day 5, followed by full-endoscopic spinal surgery/percutaneous endoscopic discectomy and drainage on hospital day 7. Blood cultures and disc aspirate cultures subsequently yielded *Prevotella denticola*. Dental computed tomography identified a periapical lesion and periodontal disease, which were considered the most likely source of bacteremia. C-reactive protein peaked at 7.64 mg/dL on hospital day 8 and gradually normalized following source control and targeted antimicrobial therapy. The patient showed clinical improvement and was transferred for rehabilitation. ABPC/SBT, ampicillin/sulbactam; AMPC/CVA, amoxicillin/clavulanate; CEZ, cefazolin; CRP, C-reactive protein; CTRX, ceftriaxone; FESS, full-endoscopic spinal surgery.

Following surgical source control and pathogen-directed antimicrobial therapy, the patient's symptoms gradually improved. Inflammatory markers steadily decreased, with CRP normalizing by hospital day 33. Approximately three weeks after initiation of pathogen-directed antimicrobial therapy, the patient became symptom-free and was subsequently transferred to a rehabilitation facility with significant clinical improvement (Figure 4).

## Discussion

This case illustrates several clinically important features. 

First,* P. denticola *was isolated from both blood cultures and disc aspirate cultures, strongly supporting its role as the true causative pathogen rather than a contaminant. Identification of the same anaerobic organism from independent specimens provides compelling microbiological evidence for hematogenous vertebral infection. 

Second, dental imaging demonstrated a periapical lesion and chronic periodontal disease. *Prevotella* species are well-recognized pathogens in odontogenic infections and are frequently recovered from periodontal pockets and periapical lesions [6-8]. Although definitive proof of the infectious source was not possible, the combination of oral pathology and bacteremia strongly suggests an odontogenic origin [5]. 

Third, this patient lacked many traditional risk factors associated with vertebral osteomyelitis, including diabetes mellitus and immunosuppression. This observation suggests that chronic oral infection alone may be sufficient to produce clinically significant bacteremia and subsequent deep-seated infection. 

The diagnosis was initially complicated by the patient's recent history of falls and trauma. In older adults, persistent back pain following trauma may lead clinicians to focus primarily on musculoskeletal injury. However, elevated inflammatory markers and progressive symptoms should prompt consideration of infectious etiologies, including vertebral osteomyelitis and epidural abscess [1-3]. 

Another notable aspect of this case was the use of FESS/PEDD. This minimally invasive technique allowed acquisition of microbiological specimens, decompression of the infected disc space, and extensive irrigation while avoiding the morbidity associated with open surgery. Combined surgical and antimicrobial management contributed to favorable clinical outcomes. 

The present case emphasizes the importance of investigating odontogenic sources whenever anaerobic bacteria are recovered from blood cultures or spinal specimens [6-8]. Collaboration between infectious disease physicians, spine surgeons, and dental specialists may facilitate the identification of occult sources and reduce the risk of recurrence.

## Conclusions

We describe a case of bacteremic spondylodiscitis and epidural abscess caused by *P. denticola* in a patient with a radiographically confirmed periapical lesion. Isolation of the organism from both blood cultures and disc aspirate cultures strongly supported hematogenous dissemination from an odontogenic source. Clinicians should consider oral infections as potential sources of anaerobic vertebral infections and pursue dental evaluation when appropriate. Early diagnosis, microbiological confirmation, source control through FESS/PEDD, and targeted antimicrobial therapy resulted in a favorable clinical outcome. 
